# Development and validation of a behaviour change intervention package to improve health literacy on behavioural risk factors of non-communicable diseases among health care assistants of government hospitals in Sri Lanka - exploratory research

**DOI:** 10.1186/s12889-025-26177-4

**Published:** 2026-01-05

**Authors:** Irshad Mashood, Dulani Samaranayake, Vindya Kumarapeli

**Affiliations:** 1 NCD, Lifestyle, Social Media and Community Health Promotion Unit, Health Promotion Bureau, Ministry of Health, Colombo, Sri Lanka; 2 Department of Community Medicine, Faculty of Medicine, University of Colombo, Colombo, Sri Lanka; 3 Directorate of Policy Analysis and Development, Ministry of Health, Colombo, Sri Lanka

**Keywords:** Health literacy, Behaviour change intervention, Non-communicable diseases, Behavioural risk factors, Healthcare assistants, Intervention mapping, Preventive health, Curriculum development, Workplace health promotion, Low- and middle-income countries

## Abstract

**Background:**

Non-communicable diseases (NCDs) are the leading cause of premature deaths globally, largely driven by modifiable behavioural risk factors such as unhealthy diet, physical inactivity, tobacco use, and alcohol consumption. Health literacy (HL) plays a vital role in modifying these behaviours. Evidence shows that Healthcare Assistants (HCAs) in Sri Lanka despite working in hospitals often demonstrate limited HL and high rates of risk behaviours. Enhancing HL among HCAs is therefore essential both for their own health and for enabling them to serve as credible health advocates in NCD prevention.

**Objective:**

This study aimed to develop and validate a Behaviour Change Intervention Package (BCIP) to improve HL related to NCD behavioural risk factors among HCAs in government hospitals in Sri Lanka.

**Methods:**

A Behaviour Change Intervention Package (BCIP) was developed using the Intervention Mapping (IM) approach, informed by the Calgary Charter HL framework. Steps included assessment of the logic model of the problem, setting objectives, intervention design, expert content validation, pilot testing, and planning for implementation and evaluation. The BCIP comprised a curriculum, facilitator guide, participant handbook, and PowerPoint presentations, for 16 two-hour sessions across eight weeks. Sessions employed lectures, role-play, group discussions, brainstorming, and m-health tools. Content validity was assessed by a 10-member expert panel, while pilot testing in selected hospitals evaluated feasibility and acceptability.

**Results:**

Findings revealed that HL among HCAs was limited by factors at individual, family, organizational, community, and policy levels. The BCIP addressed these determinants by focusing on HL’s four domains- finding, understanding, comparing, and applying health information. Expert review confirmed high relevance and appropriateness (mean scores > 3.0), while pilot testing showed feasibility and participant satisfaction with content, delivery methods, and session duration.

**Conclusion:**

The validated BCIP provides a structured, theory-driven approach to improving HL and reducing NCD risk behaviours among HCAs. Pilot findings support its feasibility for integration into routine induction or in-service training. Future studies will evaluate its effectiveness, with potential adaptation for broader workplace health promotion in Sri Lanka.

**Supplementary Information:**

The online version contains supplementary material available at 10.1186/s12889-025-26177-4.

## Background

Non-communicable diseases (NCDs) cause most premature deaths globally, driven by modifiable behavioural risk factors such as unhealthy diet, physical inactivity, tobacco use, and alcohol consumption [1]. Health Literacy (HL) related to behavioural risk factors of NCDs plays a crucial role in addressing unhealthy lifestyles that predispose to these conditions [2]. Evidence shows that the prevalence and consequences of NCDs are higher among individuals with limited HL [1]. Moreover, poorer health outcomes and increased healthcare costs associated with NCDs are frequently observed in populations with low HL [3].

The concept of HL has evolved beyond basic health education to encompass the knowledge, skills, and motivation required to access, understand, evaluate, and apply health information in decision-making [4]. Models proposed by Nutbeam and Sørensen highlight HL as both an individual capacity and a socially embedded outcome influenced by community and organizational contexts [5, 6]. The Calgary Charter further advances HL as a behavioural competency framework, emphasizing progressive skill development from accessing information to applying it confidently in daily health practices [7].

The global shift toward prevention-oriented health systems and the Sustainable Development Goals reinforce HL as a strategic priority for reducing the burden of NCDs [8]. International initiatives such as the Canyon Ranch Institute programme (CRI-LEP) and WHO’s National Health Literacy Demonstration Projects (NHLDPs) demonstrate that HL-focused interventions can lead to measurable improvements in lifestyle behaviours and health outcomes [7, 9]. However, such approaches remain limited in low- and middle-income countries, where cost-effective prevention strategies are most urgently needed.

In Sri Lanka, research on HL has primarily focused on knowledge-based assessments among specific groups such as pregnant mothers, students, teachers, and police officers, with emphasis on general health, oral health, and mental health literacy [10–12]. However, limited attention has been given to skills-based HL grounded in contemporary frameworks, and no published interventions have targeted HL related to behavioural risk factors of NCDs. Healthcare Assistants (HCAs) constitute a significant workforce in government hospitals and have frequent contact with patients. Their duties include supporting activities of daily living such as feeding, washing, dressing, mobility, and personal hygiene as well as assisting in care plan implementation under the supervision of nurses and other health professionals across hospitals, clinics, and long-term care facilities. Despite their essential role, evidence indicates that HCAs exhibit high levels of behavioural risk and low HL [13]. Given their position as potential role models and health promoters, inadequate HL not only compromises their own wellbeing but also limits their ability to influence patient behaviour and support early preventive care [14].

This critical gap highlights the need for a structured, theory-driven HL intervention targeting HCAs. Strengthening their HL has potential dual benefits: improving personal health behaviours and enabling HCAs to act as credible advocates in NCD prevention within hospital settings. Therefore, this study aimed to develop and validate a behaviour change intervention package designed to improve health literacy related to behavioural risk factors of NCDs among HCAs in government hospitals in Sri Lanka.

## Methodology

**Fig. 1:**
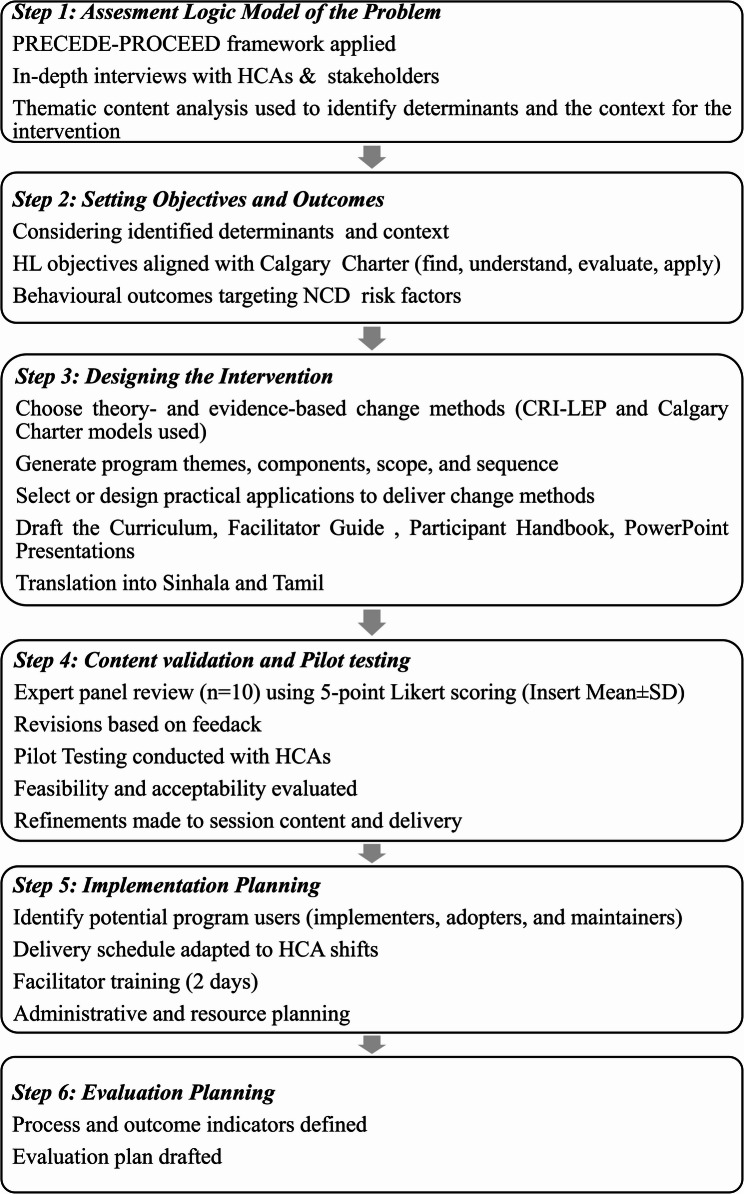
Intervention mapping steps and tasks

This Behaviour Change Intervention Package (BCIP) was developed to enhance HL related to behavioural risk factors for NCDs among HCAs. The Intervention Mapping (IM) approach guided the development process, ensuring that theoretical, empirical, and contextual evidence informed all decisions [15]. The methodological steps are presented in Fig. 1.

### Assessment of the logic model of the problem

A logic model of the problem was developed to explore determinants contributing to limited HL among HCAs. The PRECEDE-PROCEED model was applied to identify individual and environmental factors across organizational, family, community, and policy domains [16]. A qualitative study supported this assessment. In-depth interviews were conducted with 15 HCAs and additional stakeholders (hospital directors, consultants, nursing officers, overseers, and family members) in selected hospitals in the Gampaha district to explore barriers, behavioural risks, perceptions, and contextual influences. Interviews were conducted using a semi-structured interview guide (Supplementary File 1). After obtaining informed consent, interviews were audio-recorded, transcribed, and analyzed using thematic content analysis. Coding was conducted iteratively, and themes were generated to identify determinants of limited HL among HCAs. These findings informed subsequent intervention steps.

### Setting objectives and outcomes

Based on qualitative findings, HL and behavioural change objectives were formulated. The intervention aimed to improve HL across the Calgary Charter domains (find, understand, evaluate/compare, and apply health information) and reduce key behavioural risk factors: unhealthy diet, physical inactivity, tobacco use, and alcohol intake.

### Intervention design

Using evidence from existing behaviour change and HL frameworks, a comprehensive BCIP was designed. The Calgary Charter Model and elements of CRI-LEP guided session structure and content integration. The intervention incorporated interactive and participatory strategies, including group education, counselling, teamwork activities, multi-component education, social media support, and IEC materials. The intervention package consisted of four components:

1. Curriculum.
2. Facilitator guide.
3. Participant handbook.
4. PowerPoint presentation series.

All materials were developed in English and translated into Sinhala and Tamil.

### Content validation and pilot testing

Curriculum was evaluated by a 10-member expert panel, including specialists in community medicine, nutrition, psychiatry, sports medicine, and psychology. Experts reviewed the BCIP using a 5-point Likert scale to assess relevance, appropriateness, and acceptability across multiple aspects: strategies, session flow, number and duration of sessions, content of sessions and tools for three rounds of iteration. Their feedback was carefully reviewed and incorporated. The BCIP was pilot tested among HCAs in selected hospitals in Gampaha district. The package structure, content, and delivery methods were evaluated, alongside practical considerations such as administrative approvals and resource management. Participants received a full explanation and experienced the intervention as designed. Feedback was collected on all aspects including lectures, group discussions, delivery methods, and session duration using a feasibility and acceptability rating form. Based on participant ratings and comments, the BCIP was refined and finalized.

### Implementation planning

An implementation plan was developed considering resource requirements, delivery context, facilitator role, and scheduling aligned with HCAs’ shift patterns. Facilitators were required to complete a two-day orientation and training program delivered by subject experts.

### Evaluation planning

An evaluation plan was designed based on study setting (Hospital), study population (HCAs) and contents of the BCIP (HL on behavioural risk factors of NCDs).

## Results

### Determinants of limited HL among HCAs

The thematic analysis findings from the in-depth interviews are summarized in Table 1. The results indicate that limited HL arises from multiple interconnected factors operating across different socio-ecological levels. At the individual level, low educational attainment, limited communication skills, and restricted access to reliable health information were identified as major contributors to reduced HL. Family-level influences, such as spouse and parental education and traditional beliefs, further affected individuals’ understanding and decision-making. At the organizational level, barriers included limited availability of healthy options, insufficient structured health programs, and lack of ongoing support mechanisms. Community-level factors, including cultural norms, peer pressure, strong preference for curative care, and socioeconomic constraints, hindered the adoption of health-promoting behaviours. At the policy level, a narrow interpretation of HL and competing economic priorities reduced attention and investment in HL and health promotion initiatives.

**Table 1:** Determinants limited health literacy on behavioural risk factors of NCDs among HCAs

Level of influence	Factors Affecting Limited Health Literacy
Individual level	• Lower levels of formal education • Poor communication skills • Less priority given to their health and well-being in their daily routine • Limited exposure to health information • Limited access to reliable sources of health information • Overconfidence on own health • Poor motivation • Underestimation of their role. Think that HL is not a qualification of HCAs
Family level	• Lower level of parental education • Lower level of spouse education • Family Beliefs and Practices
Organizational level	• Less availability and accessibility of healthy choices • Easy availability and accessibility to unhealthy options • Exposure to traditional health awareness programs and repeated failure to implement and maintain behavior change • Seeing behavior change is a time and resource-consuming, difficult task • Limited exposure to recommended health information • Limited access to sources of recommended health information • No follow-up or evaluation after programs. Most are one-day programs • Poor frameworks and institutional structures on wellbeing
Community level	• Failure to provide an appropriate environment for learning • Conform to peers • Cultural Beliefs and Practices • Higher dependency on curative care than disease prevention and health promotion • Looking for change made by the higher levels • Attitude that responsibility for their health is on the government • Socioeconomic problems
Policy level	• Narrow understanding of HL as the ability to read or write, instead of looking at it as a skill to find, understand, compare, and apply • Economic burden and higher priority to establish economic balance instead of healthy choice

### Objectives and outcomes of the BCIP

The primary objective of the BCIP was to enhance HL across its four key domains, grounded in the Calgary Charter framework. Secondary objectives focused on reducing the prevalence of key behavioural risk factors for NCDs, specifically unhealthy dietary practices, physical inactivity, tobacco use, and alcohol consumption.

### Curriculum of the BCIP

The outline of the BCIP curriculum is shown in the Table 2. The curriculum included understanding HL concepts and definitions, analyzing its evolution, identifying NCD behavioural risk factors, applying HL principles to behavioural risk factors of NCDs, sustaining the changes achieved in beaviours and applying HL in broader health aspects. The BCIP spanned 16 two-hour sessions over 8 weeks. Delivery methods included lectures, role-playing, group discussions, Q&A, brainstorming, and m-health strategies like Whats App messages, videos, and posts. The curriculum was designed to be delivered using presentations, the facilitator guide, and the participants’ handbook to ensure consistent implementation and active engagement.

**Table 2:** Outline of the BCIP curriculum

Session	Topic	Key Learning Outcomes
1	Health Literacy – Concept, Definition & Four Domains	Understand HL concept (Calgary Charter), definition, four domains, and link between limited HL and health status
2	HL Domain 1 – Finding Recommended Health Information	Identify and locate reliable health information, apply in practical scenarios
3	HL Domain 2 – Understanding Daily/Routine Practice	Recognize and interpret routine practices in the context of health
4	HL Domain 3 – Comparing Practice vs. Recommendations	Compare current practices with recommendations, identify gaps
5	HL Domain 4 – Applying Recommendations in Daily Practice	Apply recommended health information effectively in daily life
6	Unhealthy Diet I – Salt, Sugar, Fat & Food Labels	Understand SSF risks, read labels, identify healthy diet components, know daily SSF limits
7	Unhealthy Diet II – Fruits, Vegetables, Water, Healthy Plate	Identify unhealthy plates, apply recommended intake of fruits, vegetables, and water
8	Physical Inactivity	Understand risks, barriers, and interventions for physical inactivity; prescribe suitable activity
9	Tobacco Use	Understand health & environmental harms, reasons for use, legal context, and quitting strategies
10	Alcohol Intake	Recognize health risks, alcohol types, related problems, harms to others, and interventions
11	Behaviour Change – Diet (HL 4 Domains)	Find, understand, compare, and apply healthy diet recommendations
12	Behaviour Change – Physical Activity (HL 4 Domains)	Find, understand, compare, and apply physical activity recommendations
13	Behaviour Change – Tobacco Use (HL 4 Domains)	Find, understand, compare, and apply tobacco-related recommendations (smoke & smokeless)
14	Behaviour Change – Alcohol Intake (HL 4 Domains)	Find, understand, compare, and apply alcohol-related recommendations
15	Sustainability of Behaviour Change (Mind–Body–Memory)	Link mind, memory, and body; maintain healthy habits; reinforce behaviour change
16	Applying HL to Other Health Aspects	Use HL skills to find, understand, compare, and apply recommendations in various health areas

### Content validity results

Content validation results are given in the Table 3 indicated that all components of the BCIP were rated positively by the multidisciplinary expert panel, with mean scores above 3.7 across relevance, appropriateness, and acceptability domains. Strategies used in the programme, session flow, number and duration of sessions, content of learning materials were considered relevant, appropriate, and acceptable. These findings suggest that the overall structure and content of the BCIP were viewed as suitable by experts following the iterative review process.

**Table 3:** Mean scores for BCIP by the multidisciplinary experts at the end of the third round of iteration

Item	Relevance		Appropriateness		Acceptability	
	Mean	SD	Mean	SD	Mean	SD
1. Strategies used in BCIP (lecture, role-playing, brainstorming activities, interactive group discussions with Q & A session, m-health strategies, and other information-delivering methods)	3.9	0.6	4.0	0.5	4.0	0.5
2. Flow of the sessions	3.8	0.4	3.9	0.3	3.9	0.6
3. Number of sessions (16 sessions)	3.8	0.4	4.0	0.0	3.8	0.8
4. Duration of each session (2 h per session)	3.9	0.6	3.9	0.3	3.8	0.4
5. Content of each session	3.9	0.6	4.0	0.0	3.8	0.4
6. Content of the Facilitator guide	3.9	0.6	3.9	0.3	3.8	0.4
7. Content of the Handbook for participants	3.8	0.4	3.9	0.3	3.9	0.6
8. Tools to assess outcomes	3.8	0.4	3.8	0.4	3.7	0.5

### Evaluation plan

The evaluation is planned to include both process and outcome indicators. Table 4 presents the possible process indicators, intended to assess context, reach, dose delivered, dose received, fidelity, and acceptability through observations and participant feedback. Table 5 outlines the possible outcome indicators. The primary outcome, HL level, is planned to be measured using the validated HL-NCD tool for HCAs in Sri Lanka [17]. Secondary outcomes including diet, physical activity, tobacco use, and alcohol consumption—are intended to be assessed using the WHO STEPS questionnaire, validated in Sri Lanka (2003, 2006, 2014, 2018). Baseline and post-intervention assessments are planned to evaluate changes over time.

**Table 4:** Process indicators

Aspect assessed	Indicators
Context	Level of support from hospital management to the delivery of the intervention Adequacy of dates and time allocated for the intervention from each hospital Allocation of facilities to conduct intervention from each hospital
Reach	Number of hospitals that participated in the study Number of HCAs participated in the intervention out of a planned number of HCAs to be recruited
Dose delivered	Number and percentage of hospitals where all 16 sessions were conducted Number and percentage of hospitals where each session was conducted for the planned duration
Dose received	Number and percentage of HCAs who participated in the first round in each session (16) out of the recruited HCAs Number and percentage of HCAs attended all sixteen sessions out of the recruited HCAs
Fidelity	Average of percentages on how exactly the intervention was delivered as planned (considered whether PI/trained RA related to the previous session, timing was adequate, planned activities were done, explanations were clear and the pace was acceptable when giving the average score per each session)
Acceptability	Level of satisfaction with the overall program Level of satisfaction with program content Level of satisfaction with facilitator’s skill Level of satisfaction with program length Level of satisfaction with the setting/atmosphere Level of satisfaction with program design Level of satisfaction with communication received from facilitators

**Table 5:** Outcome indicators

Aspect assessed	Indicators
Primary outcome measures	The proportion of HCAs with a Satisfactory Level of Health Literacy
Secondary outcome measures among HCAs	The proportion of HCAs consuming less than the recommended daily intake of sugar The proportion of HCAs consuming less than the recommended daily intake of salt The proportion of HCAs consuming the recommended daily intake of fruits The proportion of HCAs consuming the recommended daily intake of vegetables The proportion of HCAs with adequate levels of physical activity The proportion of HCAs using active modes of transport The proportion of ‘currently using smokeless tobacco’ among HCAs The proportion of ‘currently not smoking’ among HCAs The proportion of HCAs who are unexposed to secondhand smoking The proportion of ‘currently not consuming alcohol’ among HCAs

## Discussion

Non-communicable diseases driven by modifiable behavioural risk factors remain a major public health concern in low- and middle-income countries. In response, the BCIP was developed using HL as its theoretical foundation and guided by the IM approach. The final intervention included a curriculum, facilitator guide, participant handbook, presentation materials, and an accompanying implementation and evaluation plan.

The BCIP was developed using a systematic, theory-informed approach, addressing determinants of limited HL across multiple socio-ecological levels. At the individual level, factors such as low education, poor communication skills, and limited access to health information were identified, while family, organizational, community, and policy-level barriers further constrained HL. These findings align with previous literature highlighting the interplay between personal and environmental determinants of health behaviours [18].

The primary objective of the BCIP was to improve HL across four domains, thereby enabling HCAs to adopt healthier behaviours. Secondary objectives targeted reductions in behavioural risk factors for NCDs, echoing similar goals from interventions such as SLIMMER [19]. Consistent with the Calgary Charter, the BCIP integrated skill-building, goal setting, and supportive environments to promote sustained behaviour change, aligning with evidence from previous research on intervention development.

Designing the BCIP involved creating a culturally adapted curriculum, facilitator guide, and participant handbook, with materials translated into local languages to enhance engagement. Written resources supplemented group sessions, reflecting evidence that combined approaches are more effective in NCD interventions compared to group education alone [20].

The 16-session program, delivered over eight weeks, followed a sequential structure: five sessions introducing HL concepts, five addressing behavioural risk factors, four targeting behaviour change, and two focusing on sustainability and broader application. Sequential delivery has been found to be as effective as simultaneous approaches in multi-behaviour interventions [21]. The twice-weekly, two-hour session format fostered active engagement through role play, group discussions, and brainstorming methods supported by previous HL and lifestyle interventions [22–24]. The intervention dose and duration were informed by literature, stakeholder input, and feasibility considerations, with prior studies showing effectiveness for interventions of similar intensity and timeframe [25].

Content validity was rigorously assessed through multiple rounds of expert review, followed by pilot testing to confirm feasibility, acceptability, and understandability. This process mirrors best practices in behavioural intervention development [20]. Pilot testing played a key role in refining the BCIP. Feedback indicated that participants preferred shorter explanations and more practical demonstrations, resulting in increased use of visual aids and simplified wording in session materials. The pacing of some sessions particularly those introducing HL concepts was adjusted to allow additional discussion time. m-health components were also strengthened based on participant preference for regular reminders and short videos rather than long text messages. These refinements improved clarity, feasibility, and participant engagement.

Evaluation was planned using both process and outcome indicators, with primary outcomes being HL related to NCD behavioural risk factors measured by the validated HL-NCD tool [17] and secondary outcomes including diet, physical activity, tobacco use, secondhand smoke exposure, and alcohol intake assessed via the STEPS questionnaire. Pre- and post-tests can be scheduled two weeks before and after the intervention were intended to minimize recall bias and external influence [20].

The BCIP aligns with evidence-based principles for complex behaviour change interventions, showing careful integration of theoretical constructs, cultural adaptation, and systematic evaluation planning. Its design parallels successful HL and lifestyle programs internationally while addressing unique contextual needs of HCAs in Sri Lanka.

## Conclusion

Developed using the IM approach and guided by the Calgary Charter framework, the BCIP represents a validated behaviour change intervention designed to enhance HL and address modifiable behavioural risk factors among HCAs. The intervention shows potential to support healthier behaviours and contribute to the prevention of NCDs within this workforce. Integrating the BCIP into induction training or ongoing professional development for HCAs is recommended to strengthen both HL and health-related practices.

The programme also has potential for adaptation to other occupational groups; however, contextual modifications such as tailoring language, examples, delivery methods, and workplace health priorities would be necessary to ensure cultural and operational relevance. Collaboration with relevant authorities and stakeholders would further support feasibility, acceptability, and successful implementation in broader workplace settings.

## Supplementary Information

Supplementary Material 1.

## Data Availability

The datasets generated and analyzed during the current study are not publicly available due to participant confidentiality and ethical restrictions, but may be available from the corresponding author on reasonable request.

## Abbreviations

BCIP: Behaviour Change Intervention Package
HL: Health Literacy
NCDs: Non-Communicable Diseases
HCAs: Health Care Assistants
STEPS: STEP wise approach to Surveillance (WHO risk factor questionnaire)
HCA: Health Care Assistant
BCIP: Behaviour Change Intervention Package
HL-NCD: Health Literacy for Non-Communicable Diseases tool

## References

[1] Oo WM, Khaing W, Mya K, Moh M. Health literacy—Is it useful in prevention of behavioral risk factors of ncds? Int J Res Med Sci. 2015;3(9):2331–6. 10.18203/2320-6012.ijrms20150626.

[2] Bollars C, Sørensen K, De Vries N, Meertens R. Exploring health literacy in relation to noncommunicable diseases in samoa: a qualitative study. BMC Public Health. 2019;19(1):1151. 10.1186/s12889-019-7474-x. 31438907 10.1186/s12889-019-7474-x PMC6704563

[3] Peters RJG. Health literacy skills and the benefits of cardiovascular disease prevention. Neth Heart J. 2017;25(7–8):407–8. 10.1007/s12471-017-1001-4. 28516370 10.1007/s12471-017-1001-4 PMC5513989

[4] World Health Organization. Health literacy in the Nordic countries—Not only a determinant of health, but also a tool for health promotion. Nordic Welfare Centre. Published 1988. https://nordicwelfare.org/pub/Health_Literacy/health-literacy-concept-and-definition.html. Accessed October 29, 2024.

[5] Nutbeam D. The evolving concept of health literacy. Soc Sci Med. 2008;67(12):2072–8. 10.1016/j.socscimed.2008.09.050. 18952344 10.1016/j.socscimed.2008.09.050

[6] Sørensen K, Van Den Broucke S, Fullam J, et al. Health literacy and public health: a systematic review and integration of definitions and models. BMC Public Health. 2012;12(1):80. 10.1186/1471-2458-12-80. 22276600 10.1186/1471-2458-12-80 PMC3292515

[7] Pleasant A, Cabe J, Patel K, Cosenza J, Carmona R. Health literacy research and practice: a needed paradigm shift. Health Commun. 2015;30(12):1176–80. 10.1080/10410236.2015.1037426. 26372030 10.1080/10410236.2015.1037426

[8] World Health Organization. Shanghai declaration on promoting health in the 2030 agenda for sustainable development. Health Promot Int. 2017;32(1):7–8. 10.1093/heapro/daw103. 28180270 10.1093/heapro/daw103

[9] Bakker MM, Putrik P, Aaby A, Debussche X, Morrissey J, Borge CR, Maindal HT. Acting together – WHO National Health Literacy Demonstration Projects (NHLDPs) address health literacy needs in the European Region. Public Health Panorama. 2019;5(2):233–43. https://cris.maastrichtuniversity.nl/en/publications/acting-together-who-national-health-literacy-demonstration-project. Accessed August 18, 2025.

[10] Hettiarachchi RM, Kanthi RDFC. Oral health literacy status of pregnant mothers and effectiveness of an intervention to improve the oral health literacy of pregnant mothers in a selected MOH area in Colombo district. PGIM, University of Colombo; 2015.

[11] Mashood I, Alagiyawanne A. Health literacy and its selected associated factors among environmental Police officers in five divisions in Colombo district. PGIM, University of Colombo; 2018.

[12] Denuwara HMBH, Gunawardena NS. Level of health literacy and factors associated with it among school teachers in an education zone in Colombo, Sri Lanka. BMC Public Health. 2017;17(1):631. 10.1186/s12889-017-4543-x. 28683824 10.1186/s12889-017-4543-x PMC5500949

[13] Khargekar N, Singh A, Shruti T, Pradhan S. A cross sectional assessment of the profile of risk factors of non-communicable diseases among health care staff of a tertiary cancer hospital. J Lifestyle Med. 2022;12(2):98–103. 10.15280/jlm.2022.12.2.98. 36157886 10.15280/jlm.2022.12.2.98 PMC9490011

[14] Bosley S, Dale J. Healthcare assistants in general practice: practical and conceptual issues of skill-mix change. Br J Gen Pract. 2008;58(547):118–24. 10.3399/bjgp08X277032. 18307856 10.3399/bjgp08X277032 PMC2233962

[15] Fernandez ME, Ruiter RAC, Markham CM, Kok G. Intervention mapping: theory- and evidence-based health promotion program planning: perspective and examples. Front Public Health. 2019;7:209. 10.3389/fpubh.2019.00209. 31475126 10.3389/fpubh.2019.00209 PMC6702459

[16] Kim J, Jang J, Kim B, Lee KH. Effect of the PRECEDE-PROCEED model on health programs: a systematic review and meta-analysis. Syst Rev. 2022;11(1):213. 10.1186/s13643-022-02092-2. 36210473 10.1186/s13643-022-02092-2 PMC9549687

[17] Mashood I, Samaranayake D, Kumarapeli V. Development and validation of an instrument to assess the level of health literacy on behavioural risk factors of non-communicable diseases among health care assistants in government hospitals in Sri Lanka. J Coll Community Physicians Sri Lanka. 2025;3:45. 10.4038/jccpsl.v31i5.8864.

[18] Sabzmakan L, Jafarabadi MA, Springer A, Morowatisharifabad MA, Mohammadi E. Physical activity and healthy eating promotion among adults with cardiovascular metabolic risk factors: an application of intervention mapping framework. Health Scope. 2017;6(3). 10.5812/jhealthscope.15167.

[19] Elsman EB, Leerlooijer JN, Ter Beek J, Duijzer G, Jansen SC, Hiddink GJ, Haveman-Nies A. Using the intervention mapping protocol to develop a maintenance programme for the SLIMMER diabetes prevention intervention. BMC Public Health. 2014;14(1):1108. 10.1186/1471-2458-14-1108. 25346512 10.1186/1471-2458-14-1108 PMC4286928

[20] Taggart J, Williams A, Dennis S, Newall A, Shortus T, Zwar N, Harris MF. A systematic review of interventions in primary care to improve health literacy for chronic disease behavioral risk factors. BMC Fam Pract. 2012;13(1):49. 10.1186/1471-2296-13-49. 22656188 10.1186/1471-2296-13-49 PMC3444864

[21] James E, Freund M, Booth A, Duncan MJ, Johnson N, Short CE, Vandelanotte C. Comparative efficacy of simultaneous versus sequential multiple health behavior change interventions among adults: a systematic review of randomised trials. Prev Med. 2016;89:211–23. 10.1016/j.ypmed.2016.06.012. 27311332 10.1016/j.ypmed.2016.06.012

[22] West T, Bernhardt J. Physical activity in hospitalised stroke patients. Stroke Res Treat. 2012;2012:1–13. 10.1155/2012/813765. 10.1155/2012/813765 PMC3182066 21966599

[23] Sharp P, Spence JC, Bottorff JL, Oliffe JL, Hunt K, Vis-Dunbar M, Caperchione CM. One small step for man, one giant leap for men’s health: a meta-analysis of behaviour change interventions to increase men’s physical activity. Br J Sports Med. 2020;55(14):816–7. 10.1136/bjsports-2020-102976. 33020139 10.1136/bjsports-2020-102976

[24] Dalum P, Schaalma H, Kok G. The development of an adolescent smoking cessation intervention—an intervention mapping approach to planning. Health Educ Res. 2012;27(1):172–81. 10.1093/her/cyr044. 21730251 10.1093/her/cyr044 PMC3258281

[25] Lippke S, Corbet JM, Lange D, Parschau L, Schwarzer R. Intervention engagement moderates the dose–response relationships in a dietary intervention. Dose-Response. 2016;14(1):1559325816637515. 10.1177/1559325816637515. 27069440 10.1177/1559325816637515 PMC4811006

[26] Freebody P, Luke A. ‘Literacies’ programs debates and demands in cultural context. 1990;5(3):7–16. https://eprints.qut.edu.au/49099/. Accessed August 20, 2025.

[27] Warren-Findlow J, Coffman MJ, Thomas EV, Krinner LM. ECHO: a pilot health literacy intervention to improve hypertension self-care. HLRP Health Lit Res Pract. 2019;3(4). 10.3928/24748307-20191028-01. 10.3928/24748307-20191028-01 PMC6901363 31893258

[28] Xie B. Effects of an eHealth literacy intervention for older adults. J Med Internet Res. 2011;13(4):e90. 10.2196/jmir.1880. 22052161 10.2196/jmir.1880 PMC3222191

