# Supplementary material for: Development and validation of a behaviour change intervention package to improve health literacy on behavioural risk factors of non-communicable diseases among health care assistants of government hospitals in Sri Lanka - exploratory research
Source: BMC Public Health. 2026 Jan 5;26:441. doi: 10.1186/s12889-025-26177-4 (PMC12870722; doi:10.1186/s12889-025-26177-4)
Supplement: Supplementary file 1 — Supplementary Material 1. [file 12889_2025_26177_MOESM1_ESM.pdf]

Annexure XXI- In-depth interview guide with HCAs to develop BCIP

<u>Development of BCIP: In-Depth Interview Guide II</u>

Research title: Effectiveness of a behaviour change intervention package in improving health literacy on behavioural risk factors of non-communicable diseases among healthcare assistants of government hospitals in Colombo district- A cluster-randomized control trial.

Objective: Assessment of the logic model of the problem to develop a behaviour change intervention package (BCIP) through in-depth interviews among selected Health Care Assistants (HCA) and stakeholders.

Basic information of interview:

Name –

Age –

Sex –

Marital status –

Ethnicity –

Preferable language – Sinhala/Tamil/English

Startup session:

Greeting –

Consent –

Consensus on the venue –

Explain the objective –

Recording with consent –

Main discussion:

Area of development of BCIP	Questions (The content of the guide may be changed according to the interviewee and the flow of the interview)
**1. Perception on behavioral risk factors of NCD**	- What do you understand by the term NCD and Can you give a few examples?<br>- What do you know about the behavioral risk factors of NCD? Can you name some behavioural risk factors?<br>- Can you list out the factors which influence the behavioural risk factors of NCD of a person?
**2. Perception on Health literacy**	- What do you know about Health Literacy (HL)?<br>- How can you assess the Health Literacy among Health Care Assistants (HCA)?<br>- What is your suggestion on the questionnaire to assess Health Literacy? (Note: prompt the interviewees by giving the explanation regarding the theory of Health Literacy based on the Calgary Charter)
**3. Perception on HL-NCD (a tool developed to assess the Health Literacy on behavioural risk factors of NCD)**	- What do you know about health literacy on behavioural risk factors of NCD?<br>- What do you know about tools to assess on health literacy on behavioral risk factors of NCD?<br>- What is your opinion on the content of the questionnaire to assess Health Literacy on behavioural risk factors of NCD of a person based on the theory of Health literacy?<br>(Note: prompt the interviewees by giving the explanation on HL-NCD in relation to **Healthy Diet, Physical activity, Tobacco, and Alcohol**)
**4. Perception on associated factors of Health Literacy on behavioural risk factors of NCD among HCA**	- What are the causes for poor Health Literacy on behavioural risk factors of NCD?<br>- What are the workplace policies that support the prevention and control of behavioural risk factors of NCD?<br>- What are the conducive environment in the workplace to prevent behavioural risk factors of NCD?<br>- What are the workplace norms related behavioural risk factors of NCD?<br>- What are the causes for limited HL on behavioural risk factors of NCD among HCA?

**5. Health Literacy and Behaviour change among HCA**	- What is the relationship between HL and behaviour change?<br>- How improving HL will change the behaviour of a person?<br>- How can you improve HL on behavioural risk factors of NCD among HCA through behaviour change intervention?
**6. Perception on Behaviour change intervention package**	- How can we design content of Behaviour change intervention package (BCIP), which is appropriate and relevant to HCA?<br>- What are the communication/delivery methods HCAs prefer for BCIP?<br>- How much time (duration of the package) can be allocated for the intervention and opinion on the duration of each session?<br>- What is your opinion on support from management and other staff in regard to BCIP?
**7. Perception on evaluation of Behaviour change intervention package**	- What is your opinion on pre and post-test using HL-NCD questionnaire (tool to assess health literacy on behavioural risk factors of NCD)<br>- What is your opinion on assessing secondary outcomes using tools related to diet, physical activity, tobacco and alcohol<br>- What are the suggestions can be given to address the practical issues and overcome the challenges

Winding up the session

Clarify the missing parts

Summarize the factors discussed

Thanking the interviewee

Discussions with supervisors about finding
